# Extended mining of the oil biosynthesis pathway in biofuel plant *Jatropha curcas* by combined analysis of transcriptome and gene interactome data

**DOI:** 10.1186/s12859-021-04319-w

**Published:** 2021-08-18

**Authors:** Xuan Zhang, Jing Li, Bang-Zhen Pan, Wen Chen, Maosheng Chen, Mingyong Tang, Zeng-Fu Xu, Changning Liu

**Affiliations:** 1 CAS Key Laboratory of Tropical Plant Resources and Sustainable Use, Xishuangbanna Tropical Botanical Garden, Chinese Academy of Sciences, Kunming, 650223 Yunnan China; 2 Center of Economic Botany, Core Botanical Gardens, Chinese Academy of Sciences, Menglun, 666303 Yunnan China; 3 The Innovative Academy of Seed Design, Chinese Academy of Sciences, Kunming, 650223 Yunnan China; 4 College of Life Sciences, University of Chinese Academy of Sciences, Beijing, 100049 China

**Keywords:** Extended mining, Oil biosynthesis, *Jatropha curcas*, Transcriptome, Gene interactome

## Abstract

**Background:**

*Jatropha curcas* L. is an important non-edible oilseed crop with a promising future in biodiesel production. However, little is known about the molecular biology of oil biosynthesis in this plant when compared with other established oilseed crops, resulting in the absence of agronomically improved varieties of *Jatropha*. To extensively discover the potentially novel genes and pathways associated with the oil biosynthesis in *J. curcas*, new strategy other than homology alignment is on the demand.

**Results:**

In this study, we proposed a multi-step computational framework that integrates transcriptome and gene interactome data to predict functional pathways in non-model organisms in an extended process, and applied it to study oil biosynthesis pathway in *J. curcas*. Using homologous mapping against Arabidopsis and transcriptome profile analysis, we first constructed protein–protein interaction (PPI) and co-expression networks in *J. curcas*. Then, using the homologs of Arabidopsis oil-biosynthesis-related genes as seeds, we respectively applied two algorithm models, random walk with restart (RWR) in PPI network and negative binomial distribution (NBD) in co-expression network, to further extend oil-biosynthesis-related pathways and genes in *J. curcas*. At last, using k-nearest neighbors (KNN) algorithm, the predicted genes were further classified into different sub-pathways according to their possible functional roles.

**Conclusions:**

Our method exhibited a highly efficient way of mining the extended oil biosynthesis pathway of *J. curcas*. Overall, 27 novel oil-biosynthesis-related gene candidates were predicted and further assigned to 5 sub-pathways. These findings can help better understanding of the oil biosynthesis pathway of *J. curcas*, as well as paving the way for the following *J. curcas* breeding application.

**Supplementary Information:**

The online version contains supplementary material available at 10.1186/s12859-021-04319-w.

## Background

*Jatropha curcas* L. also called “physic nuts” (a member of the Euphorbiaceae family), is a small perennial tree or large shrub, metabolites and medicinal components of which have been used to manufacture soap and medicinal materials for a long time [1, 2]. Because of its extraordinary tolerances to environmental stresses, such as drought and infertility, *J. curcas* can grow well in bad conditions, with no endangerment to food security being a non-eatable crop. In recent years, *J. curcas* attracted more attention for high potential of biofuel plantations. The oil content of *J. curcas* is around 30–45% with a high percentage of monounsaturated oleic and polyunsaturated linoleic acid [3], so that *J. curcas* can be used directly as diesel without processing. In addition, the filter-press cake from seeds is rich in protein (60–63%) as compared with soybean (45%) [4], making it a viable resource of various amino acids.

However, there are still many challenges that limit the commercial potential of *J. curcas*. First of all, the seeds of *J. curcas* contain high levels of polyunsaturated fatty acids, which negatively impact the biofuel quality. Therefore, optimizing oil composition would facilitate the improvement of the quality of jatropha biodiesel. For instance, the reduction of unsaturated fatty acids would increase oxidative stability, the decrease of free fatty acids could prevent soap formation and increase the yield of biodiesel, and the shrinkage of 18-carbon fatty acids could lower the viscosity for better atomization of biodiesel [5]. Meanwhile, how to effectively increase oil accumulation is another critical issue in the research of oil plants, which is commonly implicated with the mechanism of lipid metabolism. However, little is known about the molecular biology of this plant as compared with other well-established oilseed crops. Besides, low seed production, uneven fruit maturation, and lack of high-yield genotypes limit the availability of this crop [6]. To make it commercially viable, new cultivars need to be developed. Genetic engineering methods could play a major role in *J. curcas* crop improvement, because the scope for classical breeding is limited due to the longer breeding cycle. For this purpose, functional genomics for understanding metabolic pathways and genetic improvement is urgent in *J. curcas*.

Driven by the development of sequencing technology, large-scale molecular biological data were generated. They comprise the relatively static data on intermolecular physical interactions, such as PPI data, as well as the quite dynamic data collected for studying gene activation during development, such as gene expression profile. Network science is gradually altering our view of cell biology by offering unforeseen possibilities to understand the internal organization of a cell [7]. Co-expression network analysis is a powerful method to extract functional modules from co-expressed genes, analyze their biological meanings, and identify important novel genes [8]. PPI network also represents strong interactions. Based on the primary roles of proteins in biological function, their interactions determine molecular and cellular mechanisms, which control healthy and diseased states in organisms. Combination of transcriptome and gene interactome data was successfully applied for efficient mining of key pathways [9, 10].

Despite many progresses achieved in genomic and transcriptomic studies in *J. curcas*, especially the information of gene expression profiles that can provide a fundamental molecular understanding of fatty acid biosynthesis, the regulatory mechanisms controlling seed development and oil biosynthesis in *J. curcas* are not very clear. In general, the process of oil biosynthesis share some similar elements among oilseed plants, therefore, the identification of these oil-biosynthesis-related genes is mostly based on BLAST hits or domain homology methods. However, *J. curcas* seeds differ greatly from other oilseed plants in terms of their oil content and fatty acid composition. Therefore, a systemic identification and analysis of the specific oil-biosynthesis- related genes of *J. curcas* are needed.

In this study, we described a multi-step computational framework for extensively mining novel oil-biosynthesis-related genes and pathways in *J. curcas* using transcriptome and gene interactome data. At first, PPI and co-expression networks in *J. curcas* were constructed using homologous mapping against Arabidopsis and transcriptome profile analysis, and further validated by network structure parameters and GO annotation consistency. We then trained the RWR algorithm on the PPI network and NBD algorithm on the co-expression network respectively, and predicted the oil-biosynthesis-related genes in *J. curcas* using the homologs of Arabidopsis genes as seeds. As a result, 27 novel oil-biosynthesis-related gene candidates were predicted. Consistent with other researches, most of the predictions exhibited high expression levels in seed development. At last, using the KNN algorithm, these genes were assigned to 5 sub-pathways, such as fatty acid synthesis and triacylglycerol biosynthesis. All these above results have shown that our proposed multi-step computational framework is a highly efficient way to mine functional pathways in non-model organisms, and these findings can help better understanding the oil biosynthesis pathway of *J. curcas*, as well as paving the way for the following *J. curcas* breeding application.

## Results

### The workflow of the key pathway extended mining algorithm

**Fig. 1:**
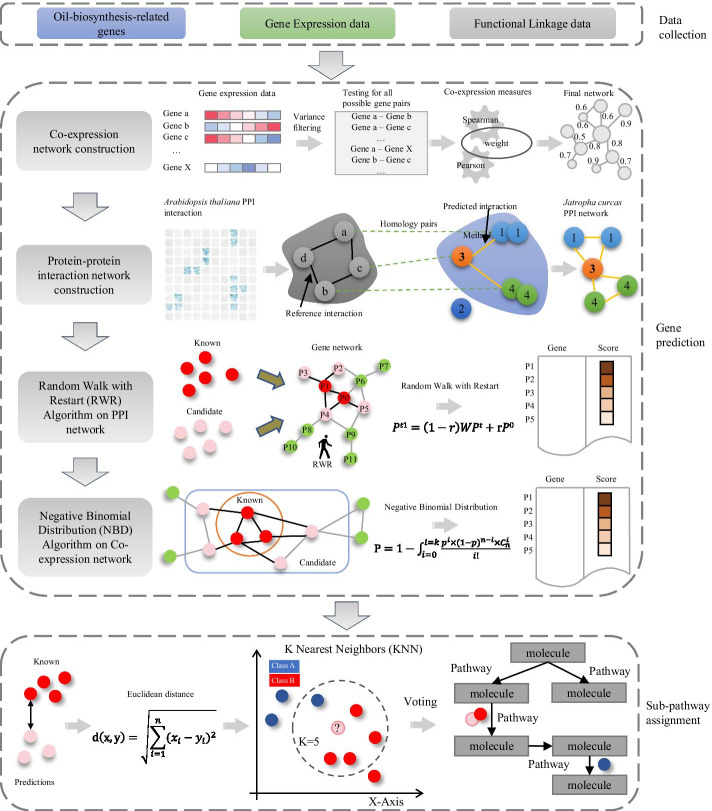
The workflow of the key pathway extended mining algorithm

Here, we designed a multi-step computational framework that integrates transcriptome and gene interactome data to mine functional pathways in non-model organisms in an extended process. The framework mainly includes three parts: data collection, gene prediction, and sub-pathway assignment (Fig. 1).

In the data retrieved part, the known oil-biosynthesis-related genes were collected from the experimentally verified oil metabolism pathways in the model species. Gene expression data was obtained from high-throughput gene expression profiling technologies such as RNA-seq or Microarray. Another widely used functional linkage data is PPI that can be collected from the STRING [11] database.

In the gene prediction part, we first constructed PPI and co-expression networks in *J. curcas*. The reference PPI was driven from high reliable *Arabidopsis thaliana* data. We inferred the PPI of *J. curcas* based on a homologous-group-based method. The gene co-expression was measured by the Spearman or Pearson correlation coefficients based on RNA-seq or Microarray expression profiles [12, 13]. As our expression profile was RNA-Seq type, Spearman correlation was selected to generate an association matrix. Then according to the different properties of the network, we respectively applied two algorithm models, RWR in PPI network and NBD in co-expression network, to predict oil-biosynthesis-related pathways and genes in *J. curcas*.

In the sub-pathway assignment part, we further classified the predicted genes into different sub-pathways according to their possible functional roles. The Euclidean distance was used to measure the distances between a candidate and all known oil-biosynthesis-related genes. Then, KNN voting method is used to assign each predicted gene to the corresponding sub-pathway.

### Data retrieved and network construction

#### Oil-biosynthesis-related gene in *J. curcas*

**Fig. 2:**
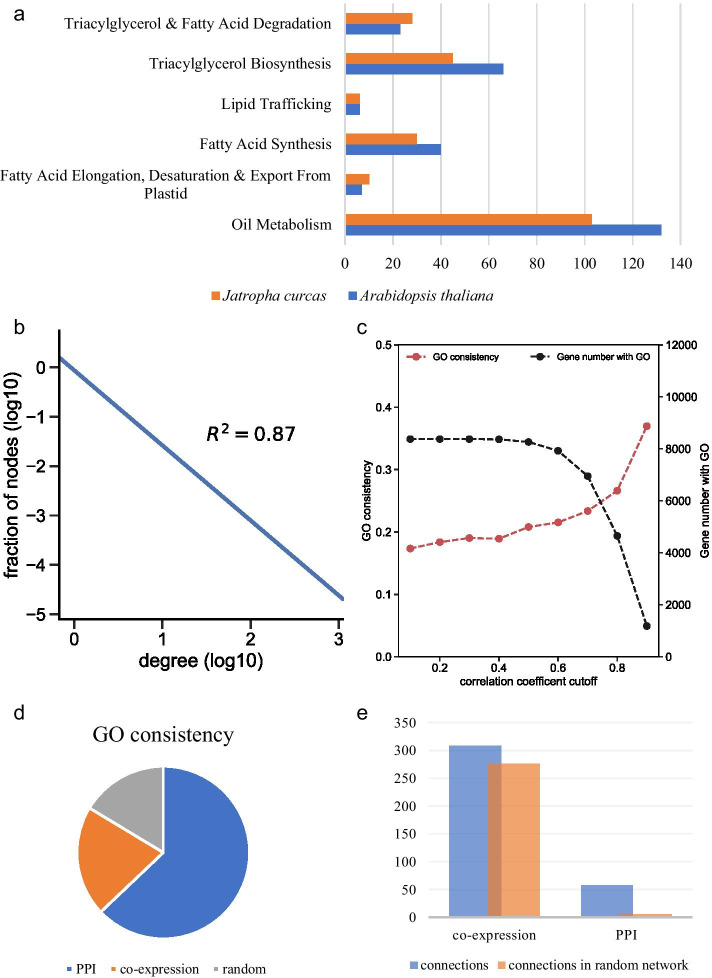
Data retrieved and network construction. **a** Comparison of the numbers of oil-biosynthesis-related homologous genes between *Arabidopsis thaliana* and *Jatropha curcas* in different pathways. **b** The PPI network of *J. curcas* conforms to a power-law distribution. **c** The change of correlation coefficient threshold and its corresponding GO consistency and number of genes with GO annotation in the co-expression network. **d** GO consistency analysis of PPI, co-expression, and random network. **e** Comparison of the numbers of connections between known oil-biosynthesis-related genes in co-expression, PPI, and random network

To obtain the whole picture of the oil synthesis pathway, we downloaded 132 Arabidopsis oil synthesis genes from ARALIP (Additional file 1). According to ARALIP, Arabidopsis thaliana oil-biosynthesis-related genes were divided into 5 sub-pathways, 40 in Fatty Acid Synthesis, 7 in Fatty Acid Elongation & Desaturation & Export From Plastid, 6 in Lipid Trafficking, 66 in Triacylglycerol Biosynthesis, and 23 in Triacylglycerol & Fatty Acid Degradation. We observed that some pathways overlapped with others. Though homology-based method, 105 oil-biosynthesis-related genes were identified as known oil metabolism genes in *J. curcas* (Additional file 2), 30 in Fatty Acid Synthesis, 10 in Fatty Acid Elongation & Desaturation & Export From Plastid, 6 in Lipid Trafficking, 45 in Triacylglycerol Biosynthesis, and 28 in Triacylglycerol & Fatty Acid Degradation. Figure 2a shows that *J. curcas*’s known oil metabolism genes account for 75% of Arabidopsis oil metabolism genes. Fatty Acid Synthesis and Triacylglycerol Biosynthesis related genes in *J. curcas* were less than Arabidopsis (75% and 68.18%) while the opposite situation was observed in Fatty Acid Elongation & Desaturation & Export From Plastid and Triacylglycerol & Fatty Acid Degradation (144.86% and 121.74%). Moreover, for the Lipid Trafficking sub-pathway, the two species have the same gene number. The detailed statistic of gene number in each sub-pathway of the two species can be found in Additional file 3. These results indicated that the core lipid metabolic pathways in the two species are carried out by a comparable number of orthologous proteins. However, an inconsistent number of genes in some pathways also indicate that there are different oil synthesis pathways between *J. curcas* and *Arabidopsis thaliana*.

#### Construction of the protein–protein network

There are 22,446 coding genes in TAIR (version 10), of which 14,051 genes can find 15,936 homologous in the *J. curcas* genome by inparinoid v4.1 (default parameters, see method). We have retrieved a very reliable Arabidopsis PPI network from literature and databases, giving a total of 17,894 Arabidopsis genes and 252,401 interactions. Through the homology-group-based method, we have finally produced the PPI network of *J. curcas* which containing 9602 nodes and 118,839 edges. For oil-biosynthesis-related genes in *J. curcas*, 86 of them are in the PPI network while 19 are not. We next analyzed the network topological characteristic of *J. curcas* PPI network. The node’s degree exhibits a power-law distribution (Fig. 2b). The scale-free R^2^ value is 0.89 and scale-free gamma is 1.52. More detailed network topological characteristics statistics can be found in Additional file 4.

#### Construction of co-expression network

There are 25,297 genes and 114 samples in the *J. curcas* expression profile. To construct a co-expression network, a suitable Spearman’s correlation coefficient (SCC) cut-off value is needed. Figure 2c shows a negative correlation between gene number with GO and SCC cut-off. At about 0.6, the network gene number with GO began to drop rapidly. We need to keep the functional genes in the network as much as possible. Our results show that 102 (97%), 91 (86%), 53 (50%), and 10 (9%) functional genes were retained by using SCC cutoff 0.6, 0.7, 0.8 and 0.9 on co-expression network, respectively (Additional file 5). So, the SCC cut-off value of 0.6 was then selected to screen significant co-expression correlations from large-scale expression data sets. Our final co-expression network consists of 22,749 nodes, 19,739,995 edges. The scale-free R^2^ value is 0.59 and scale-free gamma is 0.60. More detailed network topological characteristics statistics can be found in Additional file 6. From the above data, it’s clear that the co-expression network includes more genes in the network than the PPI network while with more noise.

#### Network validation

To verify the reliability of our networks, we used the GO consistency test based on GO enrichment analysis [14, 15]. As it can be seen in Fig. 2d, both PPI and co-expression network have much higher GO consistency values than random networks. PPI network reached 0.65, followed by co-expression network 0.22 and random network 0.17 (Fig. 2d and Additional file 7). We need to mention that the GO consistency value in the co-expression network is positively correlated with the correlation coefficient cutoff value. This indicates that GO consistency can be used as a standard to measure co-expression network reliability (Fig. 2c).

Besides, we checked if the known oil-biosynthesis-related genes are more closely connected than randomly selected nodes in PPI and co-expression networks. Figure 2e shows that the number of interactions among known oil-biosynthesis-related genes is much larger than the random set in both co-expression network and PPI network (308 vs 275.58 and 58 vs 5.8, P value 0.02 and 0, respectively). The detailed data can be found in Additional file 8.

### Prediction of oil-biosynthesis-related genes and pathway of J. curcas in PPI and co-expression networks

**Fig. 3:**
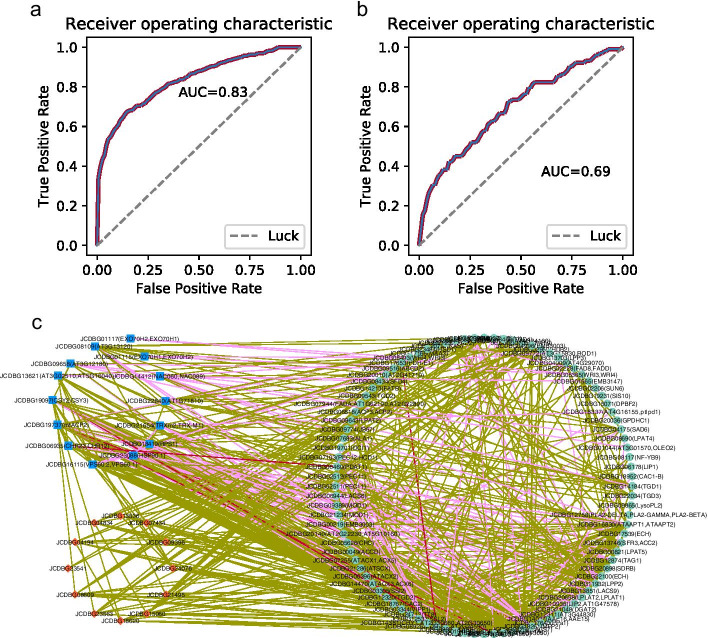
Prediction of oil-biosynthesis-related genes in PPI and co-expression networks. **a** The ROC curve of the RWR algorithm on the PPI network by leave-one-out cross-validation. **b** The ROC curve of the Negative binomial distribution method on the co-expression network by leave-one-out cross-validation. **c** Predicted oil-biosynthesis-related gene network, green node: known oil-biosynthesis-related genes; red node: oil-biosynthesis-related candidate genes predicted by Negative binomial distribution algorithm on co-expression network; blue node: oil-biosynthesis-related candidate genes predicted by RWR algorithm on PPI network; brown border: co-expression; pink border: PPI; red border: both co-expression and PPI

Because of the different topological characteristics of the co-expression network and PPI network, two different algorithms, NBD and RWR, were applied. We used leave-one-out cross-validation to evaluate the accuracy of our methods. The average area under the ROC (Receiver operating characteristic) curve (AUC) reached 0.83 by the RWR algorithm on the PPI network (Fig. 3a). On the other hand, a 0.69 AUC score was obtained by the NBD method on the co-expression network (Fig. 3b). As the value of SCC was chosen more strictly, the AUC results were correspondingly higher (Additional file 5).

Next, we predict oil-biosynthesis-related genes by RWR and NBD methods. Of the 9602 genes in the PPI network, 86 are known to be oil-biosynthesis-related and 9516 are unknown. Using the RWR possibility P > 0.001 as the threshold, we selected the top 14 candidate genes that are most closely linked to the known oil-biosynthesis-related genes (Additional file 9). Among them, gene JCDBG19737 (mtACP2), which ranks first, is the most attractive. JCDBG19737 encodes a member of the mitochondrial acyl carrier protein (ACP) family. As part of the mitochondrial matrix, it is likely to be involved in fatty acid or lipoic acid biogenesis. Although JCDBG19737 is less homology from known Arabidopsis oil-biosynthesis-related genes, RWR algorithm shows that it is more likely to have direct interaction with known oil-biosynthesis-related genes in the PPI network of *J. curcas*. Another example is gene JCDBG21654 (TRX-M1, TRXm2), which encodes m-type thioredoxin (Trx-m1), a redox activated co-chaperone, localized in the chloroplast stroma. We know that the important process of oil synthesis lies in the plastid, which may suggest JCDBG21654 is an important regulatory gene.

In the co-expression network, we predicted the candidate genes related to oil biosynthesis by calculating the possibility of each function unknown gene connecting with the known oil-biosynthesis-related genes using NBD method. As a result, 13 oil-biosynthesis-related candidate genes were predicted using p value < 0.01 as a cutoff (Additional file 9). The gene annotation indicates that they are participate in different pathways, such as JCDBG23541 is a cytochrome P450 78A7-like gene, and JCDBG13536 is a pseudogene. The known oil-biosynthesis-related genes and predicted genes by RWR and NBD methods together constitute an oil-biosynthesis-related gene network of 122 genes and 659 connections (Fig. 3c).

### The extended oil-biosynthesis-related pathway of J. curcas

**Fig. 4:**
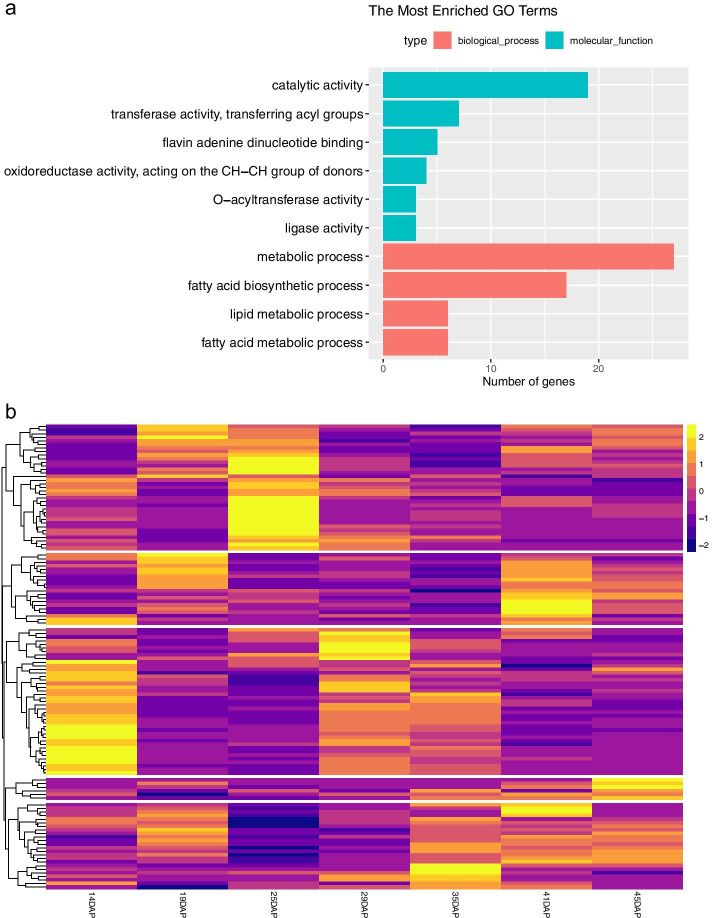
GO enrichment analysis and gene expression clustering of predicted oil-biosynthesis-related gene. **a** GO enrichment analysis of *J. curcas* predicted oil-biosynthesis-related genes. **b** Gene expression clustering of predicted oil-biosynthesis-related gene at different time points after pollination (The expression value was normalized by z-score)

Next, we studied the extended oil pathway of *J. curcas*. The GO enrichment analysis shows that the most enriched GO terms are highly related to the oil pathway (Top 10 were collected, Fig. 4a). The most enriched biological process is the metabolic process, fatty acid biosynthetic process, lipid metabolic process, and fatty acid metabolic process. The most enriched molecular function is catalytic activity, transferase activity (transferring acyl groups), flavin adenine dinucleotide binding, oxidoreductase activity (acting on the CH-CH group of donors), O-acyltransferase activity and ligase activity (Additional file 10).

Also, we did gene expression clustering analysis of predicted oil-biosynthesis-related genes at different time points of developing *J. curcas* seeds (14, 19, 25, 29, 35, 41, and 45 days after pollination (DAP). The expression matrix was download from JCDB and normalized by z-score method. Five clusters were obtained by hierarchical clustering (Fig. 4b and Additional file 11). In these five clusters, Cluster 3 exhibited the highest expression at 14 DAP and 19 DAP, suggesting that they may play an important role in lipid accumulation; Cluster 1 has a higher expression at 25 DAP while cluster 2 has a higher expression at 41 DAP; Cluster 5 continues to be highly expressed in the later stage.

**Fig. 5:**
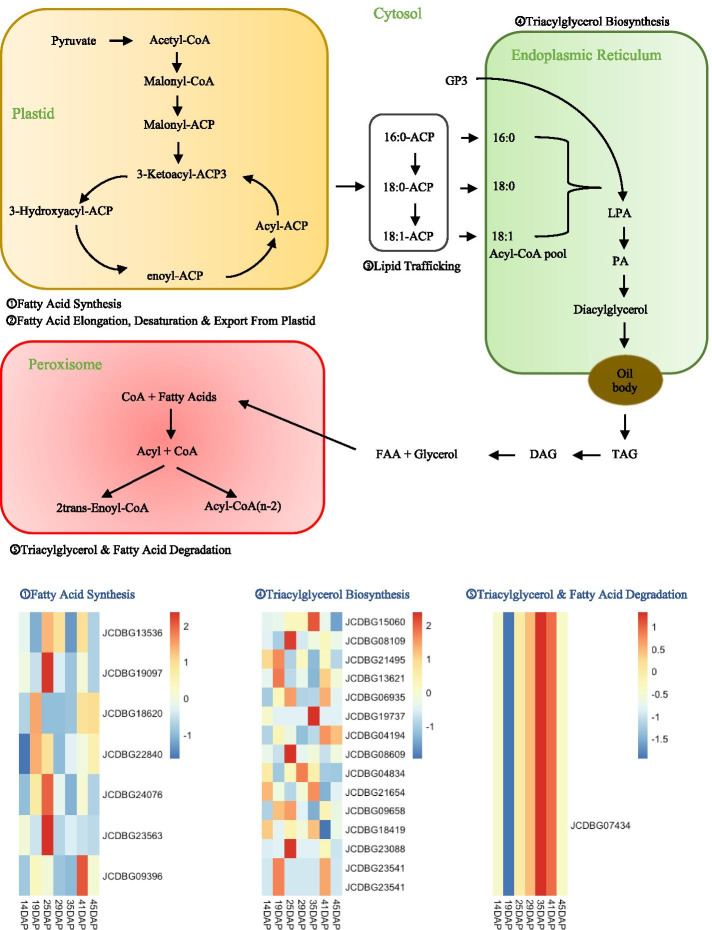
The extended oil-biosynthesis-related pathway of *J. curcas* and the gene expression profiles and potential functional roles of predicted oil-biosynthesis-related genes. ACP, acyl carrier protein; G3P, glycerol-3-phosphate; LPA, lysophosphatidic acid; PA, phosphatidic acid; TAG, triacylglycerol; DAG, dihydroxyacetone

Plant lipids are synthesized as triacylglycerols (TAGs) via a complex series of pathways in which many fatty acid (FA) biosynthetic enzymes are involved. The major FAs in plant oils are palmitic (16:0), stearic (18:0), oleic (18:1), linoleic (18:2) and linolenic acids (18:3). Among them, palmitic and stearic acids are saturated, oleic acid is monounsaturated, and linoleic and oleic acids are polyunsaturated FAs. To further study the function of our predictions, we use the KNN method to assign them into different sub-pathway – ① Fatty Acid Synthesis, ② Fatty Acid Elongation, Desaturation & Export From Plastid, ③ Lipid Trafficking, ④ Triacylglycerol Biosynthesis, and ⑤ Triacylglycerol and Fatty Acid Degradation. KNN results (Fig. 5, see Additional file 12 for detailed data) showed the oil-biosynthesis pathway with our newly predicted oil-biosynthesis-related genes, of which 7 associated with Fatty Acid Synthesis, 15 associated with Triacylglycerol Biosynthesis, and 1 associated with Triacylglycerol & Fatty Acid Degradation. The gene expression profiles of novel Fatty Acid Synthesis and Triacylglycerol Biosynthesis related genes was also shown in Fig. 5, indicating that these genes are involved in the whole process of oil biosynthesis.

## Discussion

Studies on the regulatory pathways of oil biosynthesis have great theoretical and practical value in *J. curcas.* These pathways usually involve many genes and intricate regulatory networks, and, any abnomal change in the networks would affect the whole oil synthesis, such as oil content and component diversity. However, in *J. curcas,* the regulatory pathways of oil biosynthesis are still unclear due to data deficiency and technical limitations. To the best of our knowledge, only differential expression information of developing seeds has been provided by transcriptome analysis till now. Here, we provided a systematic approach to deeply mine the oil-synthesis-related genes and pathways in *J. curcas.* The result of present study represents the first method that combined transcriptome and gene interactome data analysis of *J. curcas* and can provide insight into the biosynthesis of oil including specific triglycerides, which will contribute to the genetic improvement of *J. curcas* in seed development and oil accumulation.

In the functional study of identifying key pathways, lack of adequate analytical data is a common challenge for non-model species. As for *J. curcas,* although high-throughput measurement technology that is becoming cheaper and cheaper enriched the data for the functional research, it is still far from meeting the needs. Correspondingly, model plants, such as Arabidopsis, accumulated plenty of data for pathway research because of their well-established genome, fast transformation, and various mutants. Therefore, they can act as a powerful reference and provide some primary information for the study of other non-model species. In this work, to compensate for the data shortage in *J. curcas*, we exploited Arabidopsis transcriptomic data and functional networks as reference and scaffold to spot the potential genes and pathways associated with oil biosynthesis in *J*. *curcas*. By sequence alignment, we substantially found many oil-biosynthesis-related genes of *J. curcas* that were quite conservative between *J. curcas* and Arabidopsis. These highly conserved genes provide seeds for further prediction of more *J. curcas* specific oil-biosynthesis-related genes.

Due to the great difference between *J. curcas* and Arabidopsis in the process of oil biosynthesis, it is far from enough to rely on homologous analysis to find oil-biosynthesis-related genes and pathways in *J. curcas*. We need a method to systematically identify and analyze oil-biosynthesis-related genes and pathway in *J. curcas*, especially which are *J. curcas* specific. Because genes tend to be closely linked to genes with similar functions in gene interaction network, we may look for more oil-biosynthesis-related genes by studying the gene interaction networks of *J. curcas*, which are likely to be linked with known oil-biosynthesis-related genes in the network. On the other hand, network data may contain quite a lot of noise, so they should be used carefully, especially when predicting new genes. In the co-expression network, we used the negative binomial distribution algorithm to calculate the probability of each candidate gene participating in the key pathway. The predictions in this part were considered specific to the Jatropha oil pathway. Besides, it is important to emphasize the limitations of available PPI data once more. Our current knowledge about Jatropha protein interactome is neither complete nor distinct. The PPI data of *J. curcas* was derived from homology analysis and prediction based on Arabidopsis data. That is, it is not sure that how many interactions detected are true, there are false positives and negatives indeed. It is more difficult to obtain large-scale gene interactome data than large-scale genome and transcriptome data, which may be a critical problem for functional genomics research of non-model organisms in the future. Our method, which combines transcriptome and gene interactome data, may be a feasible and effective way at present. For the predicted results of this study, we will further use molecular biology experiments to verify their functions (related experiments are in progress).

## Conclusions

Understanding the oil metabolism pathway is key to promote the commercialization of *J. curcas*. In this paper, we presented a multi-step computational framework that integrates transcriptome and gene interactome data for mining oil-biosynthesis-related genes and assign them to obtain an extended pathway. The major advantage over simple homology search methods is that we can predict the function related genes which are species-specific. Our method can be used widely in key pathway studies, especially for the non-model organism.

## Materials and methods

### Data sources

The gene expression profiles were downloaded at April 2019 from the *J. curcas* database (JCDB [16], http://jcdb.liu-lab.com) which contained 114 RNA-Seq samples. JCDB is a comprehensive database of *J. curcas* that we have developed in previous studies. The expression profile was normalized by upper-quartile method [17]. Other information such as sequence and gene annotation retrieval details from JCDB can be found in Additional file 13. Oil biosynthesis related genes in *Arabidopsis thaliana* were collected from ARABIDOPSIS ACYL-LIPID METABOLISM PATHWAYS database (ARALIP, http://aralip.plantbiology.msu.edu/pathways/pathways) [18]. The PPIs in *Arabidopsis thaliana* were collected from literature [19–21] and databases (AtPID 5.0 [22], AtPIN 9.0 [23], and PAIR 3.0 [24]). The protein sequences and gene annotations of *Arabidopsis thaliana* were downloaded from The Arabidopsis Information Resource (TAIR) version 10[25].

### Annotation and homologue search

We used InParanoid [26] version 4.1 to find the orthologous relationships between *J. curcas* and *Arabidopsis thaliana* genes with default parameters*.* The protein sequences of the two species were used as inputs, and genes were assigned to homolog groups according to the relatedness which were measured in BLAST scores (cutoff = 40 bits). The confidence interval (cutoff = 0.05) was calculated by the bootstrap approach [27].

### Co-expression network construction

The genes with high expression variation (top 75% percentile) were retained to construct a co-expression network. We calculated the Spearman's correlation coefficient and its corresponding P value between the expression profiles of each gene-pair using our in-house Perl script (Available upon request). Only genes pairs with a correlation value higher than 0.6 and adjusted P value less than 0.01 were regarded as co-expressed in our network.

### Protein–protein interaction network migration

In one species, if two genes are detected as interacting protein–protein, we can infer that in another species, genes homologous to them are also considered to interact. These infered gene pairs are traditionally defined as interacting homologous genes. We used a homologous-group-based method to inferring *J. curcas* PPIs—If an Arabidopsis gene in group A interacts with an Arabidopsis gene in group B, then all the genes in group A of *J. curcas* interact with all the *J. curcas* genes in group B.

### Network topological characteristics

In network theory, a scale-free network is a kind of complex network in which most nodes in the network only connect with a few nodes, while few nodes connect with a lot of nodes. Its degree distribution follows a power law, at least asymptotically. The log–log plot of power-law distribution was line fitting using Eq. 1:

1 $${log}_{10}{\rm P}\left({\rm k}\right) \sim -\upgamma {log}_{10}k,$$

where k is the degree of a node, P is the fraction of nodes.

In biological networks, nodes represent genes, and the interconnected edges of nodes reflect the degree of correlation of expression. A subset of nodes that are closely connected to each other is a module. Within a module, highly connected genes, also known as "hub genes," are likely to have important biological functions. Metabolic, protein and gene interaction networks have been reported to exhibit scale-free behavior based on the analysis of the distribution of the number of connections of the network nodes [28]. To construct a biologically meaningful network with small world and scale-free structure, many network topological characteristics criteria were designed in the *J. curcas* tender shoot system [29]. We also calculated some network properties to reach this goal, such as number of genes, number of edges, connected components, the size of giant component, network density, average node degree, degree centrality, network heterogeneity, clustering coefficient, scale-free R2, and scale-free Gamma, using our in-house Perl script (Available upon request). For the PPI network parameters can be found in Additional file 4 and co-expression network parameters with different correlation coefficient threshold can be found in Additional file 6.

### GO consistency

To confirm the reliability of our PPI or co-expression network, we provided a GO consistency test [14, 15]. The basic idea of GO consistency is that in a reliable gene interaction network, a gene may share the same function (GO terms) with its neighbors. For each gene in the network, we performed GO enrichment analysis of its neighbor genes using GOATOOLS [30]. If the enriched GO terms overlapped with its own GO annotation, we counted it as a GO match. And the GO consistency was defined as N/M. Where N is the total GO match, and M is the total number of genes tested in the network. To simulate the random networks for comparison, genes were randomly selected from the network and the above steps were repeated 5000 times.

### Negative binomial distribution algorithm on weighted co-expression network

We assume that a novel oil-biosynthesis-related candidate gene has relatively more connections with known oil pathway genes than the random background. Connections across candidate and known oil-biosynthesis-related genes approximately follow a negative binomial distribution in networks. The probability P that a candidate gene is linked to k or more known oil-biosynthesis-related genes was calculated by Eq. 2:

2 $${\rm P}=1- {\int }_{i=0}^{{i=k}}\frac{{p}^{i}\times {(1-p)}^{n-i}\times {C}_{n}^{i}}{i!},$$

where p is the probability that a gene is linked to a known oil-biosynthesis-related gene by chance (p = number of known oil-biosynthesis-related genes / number of all genes), and n is the degree of the candidate gene in the network.

### Random walk with restart algorithm on PPI network

RWR is a ranking algorithm [31]. It simulates a random walker, either starts on a seed node or a set of seed nodes (here are known oil-biosynthesis-related genes), and moves to its immediate neighbors randomly at each step [32]. All the nodes in the graph are ranked by the probability of the random walker reaching this node. Let ${P}^{0}$ be the initial probability vector and ${P}^{t}$ be a vector in which the *i*th element holds the probability of finding the random walker at node i at step t. The probability vector at step t + 1 can be given by Eq. 3:

3 $${P}^{t1}=\left(1-{\rm r}\right){\rm W}{P}^{t}+{\rm r}{P}^{0},$$

where W is the transition matrix of the graph. r is the transition probability from node i to node j. The parameter ${\rm r} \epsilon (0, 1)$ is the restart probability. At each step, the random walker can return to seed nodes with probability r.

The connections between genes in the PPI network were transformed into the adjacency matrix. The restart probability was set to 0.8. The RWR function returns a matrix of values with only one column. These values represent the affinity score between each candidate genes and known oil-biosynthesis-related genes. The MATLAB code of the RWR function was download from http://www3.ntu.edu.sg/home/aspatra/research/Yongjin_BI2010.zip.

### K-nearest neighbor algorithm on function assignment of candidate genes

Penalized k-Nearest-Neighbor-Graph (PKNNG) was designed to evaluate the distances in gene expression datasets [33]. We used a basic distance-voting strategy to determine which sub-pathway the candidate genes should belong to. A candidate gene was classified by a plurality vote of its neighbors. Given the k nearest neighbors of a gene A in a network (here we use k = 5), the naive KNN method selects the functional class that is voted for by the maximum number of neighbors, and assigns it to gene A. Gene expression data in 7 different developmental stages of *J. curcas* seeds was used to calculate the distance between the candidate gene and the oil-biosynthesis-related gene. Those expression data were obtained from JCDB [16] and Jiang’s paper [34]. The distance was calculated by Euclidean distance Eq. 4:

4 $${\rm d}\left({\rm x, y}\right)=\sqrt{\sum_{i=1}^{n}{({x}_{i}-{y}_{i})}^{2}},$$

where n is the sample number of the expression data, x is the candidate gene, and y is the known oil-biosynthesis-related gene.

## Supplementary Information

**Additional file 1**. Arabidopsis oil-biosynthesis-related genes.

**Additional file 2**. *Jatropha curcas* oil-biosynthesis-related genes based on homology search.

**Additional file 3**. Homologous correspondence of oil-biosynthesis-related genes between Arabidopsis and *Jatropha curcas*.

**Additional file 4**. PPI network statistics of *Jatropha curcas*.

**Additional file 5**. The results of NBD algorithm on co-expression networks with different SCC cutoffs.

**Additional file 6**. Co-expression network statistics of *Jatropha curcas*.

**Additional file 7**. GO consistency.

**Additional file 8**. Oil-biosynthesis-related genes are closely linked relative to random background.

**Additional file 9**. Prediction of oil-biosynthesis-related genes.

**Additional file 10**. BP and MF enrichment.

**Additional file 11**. Expression clusters of predicited oil-biosynthesis-related genes.

**Additional file 12**. Predicited oil-biosynthesis-related genes in different sub-pathways.

**Additional file 13**. Retrieval details from JCDB.

## Data Availability

All datasets generated during this study are included in this published article and the sources are cited accordingly. *Jatropha curcas* gene expression profiles: http://jcdb.liu-lab.com/sdb/data/JCDB_JatCur_1.0/JCDB_1.0.gene.expression.counts.profile.zip. *Jatropha curcas* gene ontology annotation: http://jcdb.liu-lab.com/sdb/data/JCDB_JatCur_1.0/JCDB_1.0.blast2go.GO.anno.xls.zip; *Jatropha curcas* protein sequences: http://jcdb.liu-lab.com/sdb/data/JCDB_JatCur_1.0/JCDB_1.0.protein.fa.zip. *Arabidopsis thaliana* protein sequences: https://www.arabidopsis.org/download_files/Proteins/TAIR10_protein_lists/TAIR10_pep_20101214. *Arabidopsis thaliana* oil proteins: http://aralip.plantbiology.msu.edu/data/aralip_data.xlsx. The MATLAB code of the RWR function are available at http://www3.ntu.edu.sg/home/aspatra/research/Yongjin_BI2010.zip.

## Abbreviations

GO: Gene ontology
KNN: K-nearest neighbors
NBD: Negative binomial distribution
PPI: Protein–protein interaction
PKNNG: Penalized k-nearest-neighbor-graph
RWR: Random walk with restart
RNA-seq: RNA sequencing
