# Supplementary material for: Extended mining of the oil biosynthesis pathway in biofuel plant Jatropha curcas by combined analysis of transcriptome and gene interactome data
Source: BMC Bioinformatics. 2021 Aug 18;22(Suppl 6):409. doi: 10.1186/s12859-021-04319-w (PMC8375076; doi:10.1186/s12859-021-04319-w)
Supplement: Supplementary file 13 — Additional file 13. Retrieval details from JCDB. [file 12859_2021_4319_MOESM13_ESM.docx]

JCDB is a comprehensive database of J. curcas that we have developed in previous studies. All of the data we used about *J. curcas* in this study were downloaded at April 2019 from the *J. curcas* database (JCDB, http://jcdb.liu-lab.com). The protein sequences were download from http://jcdb.liu-lab.com/sdb/data/JCDB_JatCur_1.0/JCDB_1.0.protein.fa.zip; The gene ontology annotation file was download from http://jcdb.liu-lab.com/sdb/data/JCDB_JatCur_1.0/JCDB_1.0.blast2go.GO.anno.xls.zip; The expression data was download from http://jcdb.liu-lab.com/sdb/data/JCDB_JatCur_1.0/JCDB_1.0.gene.expression.counts.profile.zip.
